# Response to Battaglia and Balestracci

**DOI:** 10.1007/s00467-021-05356-6

**Published:** 2021-11-18

**Authors:** Sebastian Loos

**Affiliations:** University Medical Center Hamburg-Eppendorf, University Children’s Hospital, Martinistrasse 52, 20246 Hamburg, Germany

Dear Editors,

We thank Dr. Battaglia and Dr. Balestracci for their comments [1] on our recent article in *Pediatric Nephrology* regarding hemoconcentration and predictors in STEC-HUS [2].

Of note, 30 (26%) of their patients had mild HUS in a comparable large cohort of 116 cases (in our cohort 16/107 (15%)). In their cohort, Battaglia and Balestracci could confirm the predictive value of the score developed by Ardissino et al. [3] (hemoglobin [g/dL] + 2 × creatinine [mg/dL]) for complicated vs. uncomplicated HUS with a similar AUC of 0.86 (0.87 in our cohort). In addition, they tested the predictive value of this score for severe HUS (AUC 0.86). In our study, we chose not to include the latter analysis since we wanted to exclude a statistical bias due to the fact that creatinine is included in both the score and the end point.

With the score hemoglobin [g/dL] × LDH [U/L] we aimed to assess the predictive value of a score based on hemoglobin (reflecting hemoconcentration) and LDH (reflecting hemolysis), which is independent of kidney function. In our cohort, AUC was 0.82 for severe vs. mild HUS (AUC 0.72 in the cohort of Battaglia and Balestracci) and 0.80 (AUC 0.70 in the cohort of Battaglia and Balestracci) for complicated vs. uncomplicated HUS. From a pathophysiological standpoint, we think that it is interesting that hemoconcentration and hemolysis were fairly predictive in HUS. The weaker performance of hemoglobin × LDH in the cohort of Battaglia and Balestracci shows that such scores need to be tested in several large, ideally prospective, patient cohorts for validation.

In summary, we agree with Battaglia and Balestracci that hemoglobin + 2 × creatinine is clearly superior to hemoglobin × LDH in prediction of complicated HUS. In our opinion, this score is useful in the initial evaluation in patients with STEC-HUS at admission and it might be an important tool to stratify patients in future studies, including addressing specific treatment effects.
